# Problematic smartphone usage in the Austrian general population: a comparative study of 2022 and 2024, mental health correlates and sociodemographic risk factors

**DOI:** 10.3389/fpubh.2025.1535074

**Published:** 2025-04-08

**Authors:** Elke Humer, Marina Zeldovich, Thomas Probst, Christoph Pieh

**Affiliations:** ^1^Department for Psychosomatic Medicine and Psychotherapy, University for Continuing Education Krems, Krems an der Donau, Austria; ^2^Faculty for Psychotherapy Science, Sigmund Freud University Vienna, Vienna, Austria; ^3^Institute of Psychology, University of Innsbruck, Innsbruck, Austria; ^4^Division of Psychotherapy, Department of Psychology, Paris Lodron University Salzburg, Salzburg, Austria

**Keywords:** depression, anxiety, insomnia, alcohol abuse, stress, smartphone usage

## Abstract

**Background:**

The increasing integration of smartphones into daily life raises concerns about potential mental health impacts associated with excessive usage. This study aimed to assess trends in smartphone usage and examine its association with mental health issues as well as to assess sociodemographic risk factors for problematic smartphone usage in the Austrian population over two periods, 2022 and 2024.

**Methods:**

Two cross-sectional online surveys were conducted with representative samples of the Austrian general population (*N* = 3,057). Sociodemographic data, smartphone usage patterns, and mental health indicators, including clinically relevant depression, anxiety, insomnia, alcohol abuse, and high stress, were collected. Problematic smartphone use was defined as usage of at least 3 h per day. Chi-squared tests and multivariable logistic regression models were applied to analyze associations.

**Results:**

Smartphone usage increased significantly from 2022 to 2024, with a higher prevalence of problematic usage observed in 2024. Higher smartphone use was associated with increased odds of mental health issues, particularly for those spending at least 3 h daily on their phones. Women, younger participants, and Vienna residents showed a higher likelihood of problematic smartphone use.

**Conclusion:**

The marked increase in smartphone usage between 2022 and 2024, along with its association with mental health issues, highlights the need for public health interventions targeting digital well-being. Specific groups, notably younger individuals, women, and urban residents, may require targeted strategies to mitigate excessive smartphone usage.

## 1 Introduction

In recent years, the integration of smartphones into everyday life has redefined personal communication, access to information, professional interactions, entertainment and even the mental landscape of modern societies (1). The COVID-19 pandemic drastically altered daily routines and accelerated digital adoption and also intensified the reliance on smartphones for socialization, work, and coping with isolation (2, 3). During the pandemic, mental health challenges surged worldwide, and Austria was among the first countries providing mental health data from the first weeks of the pandemic. Studies from this period (April 2020) document a substantial increase in anxiety, depression, insomnia and stress across the population (4), which remained at an elevated level even 6 months after the first lockdown ended (5). Symptoms of depression even further increased during the third year of the pandemic when only minimal containment efforts were in place (spring 2022) (6). Smartphones have been discussed as serving as both a mitigating tool and a potential exacerbator of these issues (1, 7). It remains uncertain whether smartphone usage has declined since the end of the pandemic. On the one hand, it could be hypothesized that pandemic-era habits, particularly in digital communication and entertainment, may have persisted. On the other hand, it could be suggested that usage patterns may have shifted back. However, conclusive evidence on these trends is lacking.

Extensive research has established links between high smartphone use and negative mental health outcomes. Various studies suggest that screen time, especially excessive use of social media and other digital communications, can exacerbate feelings of anxiety, depression, and stress (8–10). Several factors are discussed to underly this associations. These include unhealthy comparisons that negatively impact self-esteem, social media burnout from constant engagement, and decreased real-life social interactions. Additionally, users may struggle with emotional regulation due to excessive preoccupation with social media, while preexisting anxiety may drive them to social media use as a coping strategy (9). In addition to these emotional effects, studies consistently find that high smartphone use negatively impacts sleep quality (9, 11, 12). As the blue light emitted by smartphones affects the circadian rhythm, individuals who use smartphones late at night are more likely to experience sleep disturbances, insomnia, and fatigue (11). Smartphone usage has also been connected to behaviors associated with substance abuse, such as increased alcohol consumption (13). The underlying mechanisms, including smartphone use as a coping mechanism to manage feelings and enhance well-being, mirror behaviors found in substance use disorders (14). While the association is complex, studies suggest that individuals who frequently use smartphones as a means of emotion regulation are at higher risk of developing maladaptive coping behaviors (15).

The extent of smartphone use is influenced by various sociodemographic factors, such as age, gender, location, and migration background. These factors influence how individuals use and respond to digital technologies in their daily lives.

As summarized in a meta-analysis by Sohn et al. (16), the majority of studies report that women are more susceptible to problematic smartphone use then men. For women, higher smartphone use has been associated with increased social media engagement (3), which may heighten vulnerability to issues like body image concerns, cyberbullying, and social comparison, all of which can impact mental health (17–19).

Younger people, especially adolescents and young adults, are also disproportionately affected (20). The developmental period of adolescence involves heightened social sensitivity to both positive and negative social stimuli (21). Smartphone use potentially amplifies peer problems and exposure to cyberbullying or unfavorable social comparisons (22), which likely contributes to the observed association of social media use and exacerbated mental health issues like anxiety and depressive symptoms in adolescents (23).

Individuals with a migration background may develop a unique relationship with their smartphones. For some migrant groups, smartphones provide a crucial link to maintain connections with family and friends who stayed in their countries of origin (24). However, migrant populations often face challenges related to integration, discrimination, and social isolation (25), which may increase the risk of problematic smartphone use as a coping mechanism (26).

Education and income levels can also influence patterns of smartphone use and its impact on mental health. While some research suggests that individuals with lower socioeconomic status may be more prone to problematic smartphone use (27), other studies observed positive associations of family income (28) and higher economic status (29) with problematic smartphone usage.

Employment status might also impact smartphone use, although research has been inconclusive (30, 31). In general, unemployment might contribute to feelings of low self-esteem (32), potentially leading to problematic smartphone use as a distraction or coping mechanism. In contrast, employed individuals who frequently use smartphones for work-related purposes, such as answering emails outside of work hours, may experience increased stress and reduced work-life balance. This “always-on” culture can exacerbate fatigue, insomnia and anxiety, further linking smartphone use with mental health concerns (33).

Geographic factors, such as urbanization, also play a role in smartphone usage and its psychological effects. Previous studies suggest that urban dwellers tend to spend more time on their smartphones than their rural counterparts (34, 35).

The role of partnership status on smartphone use and mental health is complex. While those who are single may be more likely to use smartphones for social interaction and dating, smartphone usage might not be necessarily related to the actual availability of a partner. Individuals in a low-quality relationship might also use social media to reduce unpleasant feelings such as boredom or loneliness (36).

To deepen the understanding of the intricate links between smartphone use and mental health, it is essential to consider how different sociodemographic factors contribute to problematic smartphone usage.

This study seeks to address the gaps in understanding whether smartphone use patterns have shifted in Austria’s post pandemic population and to assess the relationship between smartphone use and mental health in Austria by analyzing survey data from representative population samples collected cross-sectionally in April 2022 and October 2024. Further, the study investigates sociodemographic factors, such as age, gender, migration background, income, and geographic location, to identify specific groups at risk for problematic smartphone use. As several sociodemographic factors are not independent of each other (e.g., higher income in persons with higher education level or higher proportion of migrants in Vienna compared to other Austrian federal states), the aim of our study to investigate the independent contribution of each sociodemographic variable in predicting the prevalence of problematic smartphone usage by adjusting for the other sociodemographic variables.

The following research questions were addressed:

1. Has smartphone usage changed in the Austrian general population from 2022 to 2024?
2. What is the relationship between smartphone usage and mental health outcomes, such as clinically relevant depression, anxiety, sleep disturbance, alcohol abuse and high stress?
3. Which population groups in Austria are at higher risk for problematic smartphone use?

## 2 Methods

### 2.1 Design and participants

Two independent cross-sectional online surveys were conducted on a representative sample of the Austrian general population according to age, gender, region, and educational level. The first survey took place between April 19 and 26, 2022 and the second survey took place between October 10 and 28, 2024. Sociodemographic characteristics of the study sample are summarized in Table 1.

**Table 1:** Study sample characteristics (*n* = 3,057)

**Variable**	*N*	**%**
**Gender**		
Male	1537	50.3
Female	1515	49.6
Diverse	5	0.2
**Age**		
14-24	292	9.6
25-34	539	17.6
35-44	543	17.8
45-54	549	18.0
55-64	605	19.8
≥65	529	17.3
**Region**		
Vienna	649	21.2
Upper Austria	493	16.1
Lower Austria	582	19.0
Carinthia	178	5.8
Styria	448	14.7
Tyrol	255	8.3
Salzburg	197	6.4
Burgenland	120	3.9
Vorarlberg	135	4.4
**Education**		
No formal education	12	0.4
Secondary school	412	13.5
Apprenticeship	1130	37.0
Vocational secondary school	548	17.9
High School	534	17.5
University	421	13.8
**Migration Background**		
Yes	410	13.4
No	2647	86.6
**Work situation**		
In employment	1876	61.4
Unemployed	414	13.5
Retired	767	25.1
**Net household income**		
< € 1000;	310	10.1
€ 1000,- to € 2000,-	764	25.0
€ 2001,- to € 3000,-	801	26.2
€ 3001,- to € 4000,-	530	17.3
> € 4000	652	21.3
**Partnership status**		
Single	981	32.1
Living in partnership	2076	67.9
**Year**		
2022	1032	33.8
2024	2025	66.2

Note: Migration background was defined as whether both parents

were born abroad (second-generation immigrants) or participants

themselves were born abroad (first-generation immigrants).

This study was conducted following the Declaration of Helsinki and approved by the Ethics Committee of the University for Continuing Education Krems, Austria (Protocol Code: EK GZ 26/2018–2021) and the Ethics Committee of the Sigmund Freud University Vienna (Protocol Code: PD92HEDOC81GDC91138; pre-registration on ClinicalTrials.gov: NCT06621537). All participants gave electronic informed consent to participate and complete the questionnaires.

### 2.2 Measures

#### 2.2.1 Sociodemographic variables

Sociodemographic information was gathered using eight key variables. Participants reported their gender (female, male, diverse), age (in years), region (federal state), highest educational level (no formal education; secondary school; apprenticeship; vocational secondary school; higher secondary school; university), monthly net household income (<€1,000; €1,001–€2000; €2001–€3,000; €3,001–€4,000; >€4,000), employment status (employed; unemployed; retired), migration background (whether the participant or both parents were born outside the country), and relationship status (single; in a partnership). For the statistical analyses, gender-diverse individuals were excluded (*n* = 5) due to the low number. Also, the categories “no formal education” and “secondary school” were combined due to a low number of participants reporting no formal schooling (*n* = 12).

#### 2.2.2 Smartphone usage

Participants were asked about their hours per day spent on their smartphone: <1, 1–2, 3–4, 5–6, 7–8, >8.

In line with previous research, problematic smartphone usage was operationalized as usage of at least 3 h per day [e.g., (37–39)]. This pragmatic cut-off has been used in large-scale population studies to indicate a threshold beyond typical daily use. However, it should be noted that this approach does not differentiate between types of smartphone use (e.g., work-related vs. recreational) or assess behavioral dependency. Future studies should consider the use of validated instruments such as the Smartphone Addiction Scale (SAS) or Mobile Phone Problem Use Scale to better capture the multidimensional nature of problematic smartphone use, including aspects of compulsive behavior, tolerance, withdrawal, and interference with daily functioning.

#### 2.2.3 Depressive symptoms (PHQ-9)

Symptoms of depression over the past 2 weeks were assessed with the German version of the Patient Health Questionnaire (PHQ-9) (37). The PHQ-9 comprises nine items that are self-rated on a four-point Likert-type scale from 0 (not at all) to 3 (nearly every day) with total scores ranging from 0 to 27. A cut-off point of at least 10 point was used in participants aged at least 18 years to categorize clinically relevant depression, whereas a cut-off of ≥11 was applied for participants aged between 14 and 17. In this sample the internal consistency (Cronbach’s alpha) was *α* = 0.89.

#### 2.2.4 Anxiety symptoms (GAD-7)

Symptoms of anxiety over the past 2 weeks were assessed with the German version of the Generalized Anxiety Disorder scale (GAD-7) (38). The GAD-7 comprises seven items that are self-rated on a four-point Likert-type scale from 0 (not at all) to 3 (nearly every day) with total scores ranging from 0 to 27. Cut-offs for identifying clinically relevant anxiety were ≥ 11 for adolescents (14–17 years) and ≥ 10 for adults. Cronbach’s alpha was *α* = 0.91 in the sample at hand.

#### 2.2.5 Insomnia symptoms (ISI-7)

Sleep quality and symptoms of insomnia were assessed with the Insomnia Severity Index (ISI-7) (39). The ISI-7 includes seven self-rated items, each scored on a five-point scale from 0 to 4. The total score can range from 0 to 28, with a score of 15 or higher indicating clinically relevant insomnia. In this sample, the internal consistency was *α* = 0.87.

#### 2.2.6 Symptoms of alcohol abuse (CAGE)

Alcohol abuse symptoms were evaluated with the German version of the CAGE (40) questionnaire. This tool includes four yes/no questions that inquire about potential signs of alcohol dependency, including efforts to cut down, annoyance with others’ criticism, feelings of guilt, and an eye-opener. The total score ranges from 0 to 4, with scores of 2 or higher suggesting clinically relevant alcohol abuse. In this sample Cronbach’s alpha was *α* = 0.66.

#### 2.2.7 High stress (PSS-4)

Subjective stress levels over the past 4 weeks were assessed using the German version of the short version of the Perceived Stress Scale (PSS-4) (41). This self-report measure evaluates perceived stress on a five-point Likert-type scale, ranging from 0 (never) to 4 (very often). The total score is calculated by summing responses, with items 2 and 3 being reverse-coded, resulting in a score range from 0 to 16. Higher scores reflect greater perceived stress, with a score of 6 or above indicating high stress levels. Cronbach’s alpha was *α* = 0.78.

### 2.3 Statistical analyses

Chi-squared tests were applied to

1. assess differences in smartphone usage between the two survey periods.
2. examine differences in the prevalence of clinically relevant depression, anxiety, insomnia, alcohol abuse and stress with smartphone usage.

Multivariable logistic regressions were applied to

1. assess the association of smartphone usage with mental health indicators while adjusting the data for gender, age and survey period with the mental health indicators being the dependent variables.
2. investigate the association of problematic smartphone usage with sociodemographic characteristics including problematic smartphone usage as dependent variable and gender, age, migration background, region, education, income, employment situation, and relationship status as predictors. The survey period was also included in the model as differences were observed in the proportion of individuals with problematic smartphone usage between both survey periods. Correlations between predictor variables were low (*r* < 0.75), indicating that multicollinearity was not a confounding factor in the analysis.

*p*-values of less than 0.05 were considered statistically significant (2-sided tests) before Bonferroni correction. To correct for multiple testing within families of related hypothesis *p*-values were adjusted to *p* < 0.008 for Chi-squared tests assessing differences in prevalences of problematic smartphone usage within age groups (0.05/6 tests). Similarly, for associations between prevalence rates of clinically relevant mental health symptoms and problematic smartphone usage a *p*-value of <0.008 was applied (0.05/6 tests). For binary logistic regression analyses investigating associations of smartphone usage with mental health indicators *p*-values were adjusted to 0.004 (0.05/12 tests). For the analysis of the association of problematic smartphone usage with sociodemographic characteristics, the p-value was set to 0.002 (0.05/27 tests).

Adjusted odds ratios (aORs) and their 95% confidence intervals (CIs) were estimated to assess the statistical uncertainty. All statistical analyses were performed using SPSS version 26 (IBM Corp, Armonk, NY, United States).

## 3 Results

### 3.1 Association of smartphone usage with survey period

**Figure 1:**
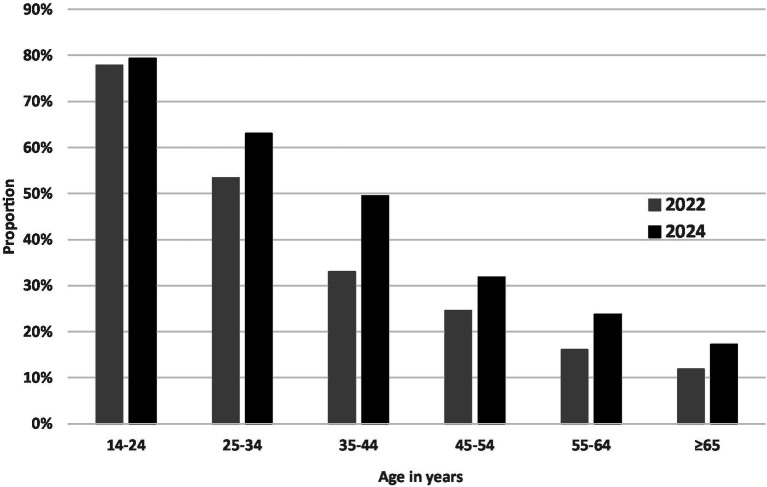
Proportion of participants spending ≥3 h/d on the smartphone by age category and survey period.

Smartphone usage increased in October 2024 compared to April 2022 (*p* = 0.011; Table 2). While the proportion of those spending less than 1 h/d on the smartphone decreased by 17.5%, the proportion of those spending more than 8 h/d on the smartphone increased by 60.7%. A more detailed analysis by age group (Figure 1) revealed that while the proportion of individuals exceeding the cut-off of 3 h/d being indicative or problematic smartphone usage did not change in the youngest age group (14 to 24 year olds), this proportion increased in persons aged 25 to 34 years by 50.0% (*p* < 0.001).

**Table 2:** Smartphone usage in April 2022 and October 2024 (n = 3,052)

Smartphone Usage	Unit	Survey period		
		2022 (*N* = 1031)	2024 (*N* = 2021)	*P*
< 1h/d	% (n)	29.7 (306)	24.5 (495)	0.011
1-2h/d	% (n)	35.8 (369)	35.7 (721)	
3-4h/d	% (n)	24.2 (71)	26.1 (528	
5-6h/d	% (n)	6.9 (71)	8.5 (172)	
7-8h/d	% (n)	1.6 (16)	2.1 (42)	
>8h/d	% (n)	1.9 (20)	3.1 (63)	

### 3.2 Association of smartphone usage with mental health indicators

**Figure 2:**
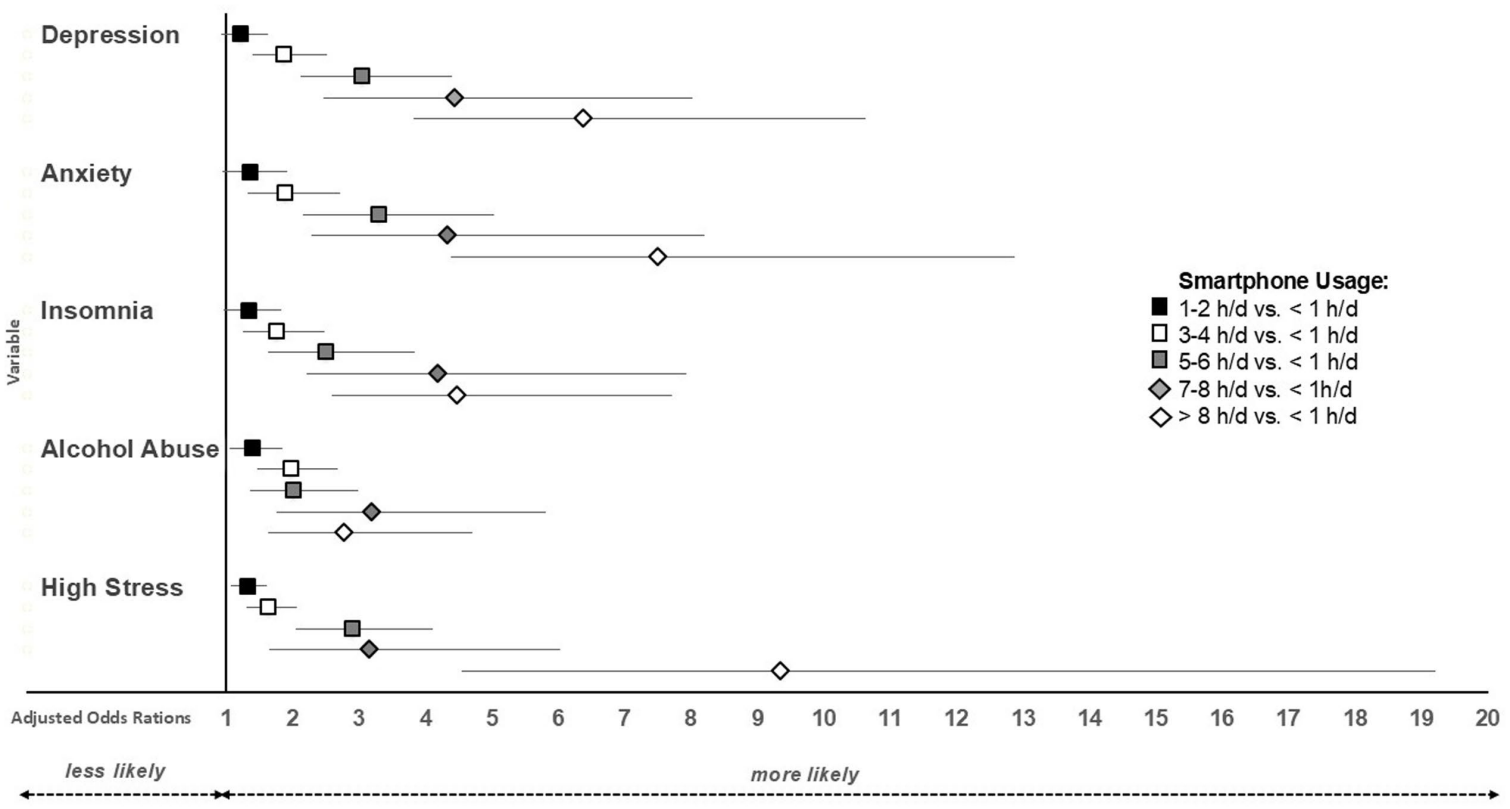
Adjusted odds ratios and their 95% confidence intervals for different smartphone usage time categories vs. <1 h smartphone usage per day.

Chi-squared tests revealed a higher prevalence of clinically relevant depression, anxiety, insomnia, alcohol abuse and high stress with increasing time spent on the smartphone (all *p* < 0.001; Table 3). When adjusting for gender, age, and the survey period, spending at least 3 h/d on the smartphone vs. <1 h/d on the smartphone increased the odds for all investigated mental health outcomes (all *p* < 0.004; Figure 2). Spending 1–2 h/d vs. <1 h/d on the smartphone did not significantly increase the odds for clinically relevant mental health symptoms (*p* > 0.004).

**Table 3:** Proportion of participants exceeding the cut-off scores for clinically relevant depression, anxiety, insomnia, alcohol abuse and high stress by smartphone usage (*n* = 3,052)

Variable	Unit	Smartphone Usage							< 1h/d (*n* = 801)	1-2h/d (*n* = 1090)	3-4h/d (*n* = 777)	5-6h/d (*n* = 243)	7-8h/d (*n* = 58)	> 8h/d (*n* = 83)	*P*
Depression	% (n)	12.5 (100)	19.0 (207)	30.8 (239)	42.8 (104)	51.7 (30)	57.8 (48)	<0.001
Anxiety	% (n)	6.7 (54)	12.0 (131)	19.2 (149)	30.0 (73)	36.2 (21)	47.0 (39)	<0.001
Insomnia	% (n)	8.9 (71)	12.8 (140)	16.5 (128)	21.4 (52)	31.0 (18)	32.5 (27)	<0.001
Alcohol Abuse	% (n)	12.1 (97)	18.4 (201)	25.2 (196)	26.3 (64)	37.9 (22)	32.5 (27)	<0.001
High Stress	% (n)	37.3 (299)	49.8 (543)	60.6 (471)	74.1 (180)	75.9 (44)	89.2 (74)	<0.001

### 3.3 Association of problematic smartphone usage with sociodemographic characteristics

Multivariable logistic regression analyses (Table 4) revealed that the likelihood of problematic smartphone usage (i.e., spending at least 3 h/d on the smartphone) was associated with gender, age, and region.

**Table 4:** Results of the multivariable binary logistic regression analyses on the association of sociodemographic factors and survey period on the odds for problematic smartphone usage

Variable	*p*-value	aOR	95% CI	
**Gender** (female vs. male)	<0.001	1.504	1.271	1.781
**Age**
25-34 years vs. <25 years	<0.001	0.369	0.259	0.524
35-44 years vs. <25 years	<0.001	0.193	0.136	0.276
45-54 years vs. <25 years	<0.001	0.097	0.068	0.140
55-64 years vs. <25 years	<0.001	0.057	0.038	0.084
≥65+ years vs. <25 years	<0.001	0.032	0.020	0.054
**Migration background** (yes vs. no)	0.136	0.831	0.652	1.060
**Education**
Apprenticeship vs. no formal/secondary education	0.630	0.938	0.721	1.219
Vocational secondary school vs. no formal/ secondary education	0.012	0.674	0.496	0.916
High school vs. no formal/ secondary education	0.007	0.655	0.482	0.889
University vs. no formal/secondary education	0.772	0.953	0.688	1.320
**Region**
Upper Austria vs. Vienna	0.007	0.686	0.523	0.901
Lower Austria vs. Vienna	0.036	0.757	0.583	0.982
Carinthia vs. Vienna	0.431	0.857	0.584	1.258
Styria vs. Vienna	0.004	0.665	0.504	0.878
Tyrol vs. Vienna	<0.001	0.537	0.381	0.756
Salzburg vs. Vienna	0.085	0.725	0.504	1.045
Burgenland vs. Vienna	0.422	0.827	0.520	1.315
Vorarlberg vs. Vienna	0.110	0.703	0.457	1.083
**Income**
€ 1000,- to € 2000,- vs. < € 1000	0.603	1.085	0.799	1.473
€ 2001,- to € 3000,- vs. < € 1000	0.481	1.118	0.819	1.527
€ 3001,- to € 4000,- vs. < € 1000	0.202	0.801	0.569	1.127
> € 4000 vs. < € 1000	0.123	0.766	0.545	1.075
**Partnerships status** (yes vs. no)	0.988	0.999	0.822	1.212
**Employment**
Unemployed vs. employed	0.195	1.184	0.917	1.529
Retired vs. employed	0.082	1.348	0.963	1.887
**Survey period (2024 vs. 2022)**	<0.001	1.676	1.396	2.011

More specifically, women were more likely than men to show problematic smartphone usage (aOR: 1.50, 95% CI: 1.27, 1.78). Furthermore, a strong association with the age group was found, with a strong decline in the aORs (aORs 0.032 to 0.37) with increasing age vs. those between 14 and 24. No association with migration background and education was observed. The analysis of potential regional differences revealed that inhabitants from Tyrol had lower odds compared to those residing in the capital Vienna (aORs 0.54). The income level and the employment situation were not associated with the likelihood of problematic smartphone usage. Furthermore, no association with the partnership status was found.

In addition to odds ratios, we calculated absolute risk differences (ARD) to assess the practical significance of group differences. Women had a 9-percentage-point higher prevalence of problematic smartphone use compared to men. Compared to participants aged between 14- and 25-years ARDs decreased by 18.8 percentage units in participants aged between 25 and 34 years, by 34.7 percentage units in participants aged between 35 and 44 years, by 49.1 percentage units in participants aged between 45 and 54 years, by 57.3 percentage units in participants aged between 55 and 64 years, and by 63.1 percentage units in participants aged 65 years or older. Residents of Vienna exhibited a 14.9-percentage-point higher prevalence than those in Tyrol.

## 4 Discussion

This study investigated the relationship between smartphone usage and mental health, focusing on the Austrian population across two survey periods, April 2022, and October 2024. The findings reveal that problematic smartphone usage (defined as usage of at least 3 h daily) was more prevalent in October 2024 than in April 2022, reflecting a concerning upward trend. Furthermore, significant associations between increased smartphone use and a heightened prevalence of mental health issues, including depression, anxiety, insomnia, alcohol abuse, and high stress, were observed. Specifically, spending at least 3 h daily on a smartphone was linked with a notably higher likelihood of these problems. Additionally, sociodemographic characteristics such as gender, age, and region were associated with problematic smartphone use, with women, younger individuals, and Vienna residents showing a higher tendency toward excessive usage.

While our findings demonstrate a significant increase in smartphone usage from 2022 to 2024, the relationship between these trends and mental health outcomes has been explored in a separate study by our team (42). The current study, therefore, focuses specifically on smartphone use patterns and their cross-sectional associations with mental health indicators.

### 4.1 Change in smartphone usage in the Austrian general population from 2022 to 2024

The observed increase in smartphone usage from April 2022 to October 2024 might be influenced by multiple global and socio-political factors. Since 2022, individuals worldwide have faced compounded crises, including the COVID-19 pandemic, economic inflation, and the escalation of international conflicts like the Ukraine war and, more recently, escalation of violence in the Middle East. These events might have intensified reliance on digital devices for information, connection, and even emotional relief. Research suggests that social media use can become a coping mechanism during times of heightened stress (43, 44).

However, while these contextual stressors are plausible explanatory factors for the observed trends, it should be noted that our study did not incorporate specific external variables (e.g., economic stress, unemployment rates, or pandemic-related restrictions) into the statistical analysis. As such, we cannot empirically assess the extent to which these broader societal influences contributed to the increase in smartphone use. Future studies should consider linking individual-level survey data with time-sensitive macroeconomic or social indicators to more accurately contextualize behavioral trends and identify interaction effects between individual and contextual risk factors.

The relative stability of high usage rates among younger individuals (14–24 years) contrasts with the sharp increases observed among adults aged 35 to 44 years. This stability may be due to a ceiling effect, as 78% of this younger age group already exhibited problematic smartphone usage times in 2022, leaving less room for further increases. In contrast, older adults, seem to adapt more recently to digital reliance. The 35–44 age group, in particular, may be facing heightened digital demands due to work expectations and family responsibilities, which now often involve managing both professional and household tasks digitally (45, 46).

### 4.2 Smartphone usage and mental health

Our results align with existing research indicating a link between excessive smartphone usage and mental health problems. Previous studies have associated high smartphone use with depression, anxiety, sleep disturbances, and stress, with underlying mechanisms potentially including digital addiction, social comparison, and reduced satisfaction with face-to-face interactions (9, 47). The association with alcohol misuse, may reflect tendencies toward unhealthy mechanisms to manage stressful situations in individuals who excessively use smartphones. A recent meta-analysis supports this notion, showing a positive association of mobile phone addiction and negative coping styles, but no association with positive coping styles (48).

It is important to note that the observed associations do not imply causation. While excessive smartphone use was linked to higher odds of depression, anxiety, insomnia, alcohol misuse, and stress, the cross-sectional nature of the study does not allow us to determine the direction of these relationships. Reverse causality is possible—individuals experiencing mental health issues may engage in higher smartphone use as a coping mechanism. Future longitudinal studies are needed to establish causal pathways and disentangle bidirectional influences.

The observed association between excessive smartphone use and increased odds of mental health issues across multiple domains underlines the pressing need for public health initiatives that address digital well-being, particularly considering the substantial increase in time spent daily on the smartphones noted between 2022 and 2024.

### 4.3 Socio-demographic correlates of problematic smartphone use

The finding that women have a higher likelihood of problematic smartphone usage is also in line with previous studies. Women may be more susceptible to smartphone overuse due to social media engagement (3, 16), which has been associated with increased mental health risks (9).

Younger individuals were far more likely to engage in problematic smartphone usage than older age groups. As “digital natives” they view smartphones as an essential part of daily life, integrating them more seamlessly into routines for socializing, entertainment, and even education or work – unlike older generations who adopted the technology later (49). As the developmental stage of adolescence is characterized by social sensitivity and identity formation (21, 50), smartphone overuse may exacerbate mental health vulnerabilities through increased exposure to online comparison and cyberbullying risks (9, 51).

Participants residing in Vienna exhibited a higher likelihood of excessive smartphone use compared to residents in Tyrol. This trend may be attributed to several urban lifestyle factors characteristic of large cities such as higher levels of social isolation (52), which can increase digital engagement as a substitute for in-person connections. Furthermore, rural areas often have less developed digital infrastructure, limiting access to high-speed internet and reducing opportunities for continuous smartphone use (35). This disparity in digital access may partially explain why smartphone dependency appears more pronounced in urban centers like Vienna.

Interestingly, no significant associations were found between problematic smartphone use and education or income levels. One possible explanation is that other, unmeasured factors—such as occupational demands, digital work integration, or lifestyle habits—may confound this relationship. Future research should explore these potential moderators in more detail to clarify the role of socioeconomic factors in smartphone use patterns.

### 4.4 Limitations

This study has several limitations that should be considered. First, the cross-sectional design of the two surveys limits the ability to establish causal relationships. It remains unclear whether excessive smartphone use contributes to mental health problems or whether individuals experiencing psychological distress are more likely to overuse their smartphones as a coping strategy. Longitudinal studies are needed to clarify these directions of influence and to explore potential mediating factors.

Additionally, the exclusion of gender-diverse individuals due to their small representation in the sample limits the generalizability of findings to all gender identities. Future research should include larger samples of gender-diverse individuals to allow for a more comprehensive analysis of gender-related differences in smartphone use and mental health outcomes.

Another limitation lies in the use of a time-based cut-off (3 h daily) to define problematic smartphone use. Although supported by prior research, this approach may oversimplify the complex behavioral patterns associated with smartphone addiction and does not differentiate between productive and non-productive use. This approach may misclassify individuals who use smartphones for work or productivity purposes as problematic users, while underestimating compulsive use in individuals who spend less time on their phones but exhibit addiction-like behaviors. Future studies should apply validated psychometric tools such as the Smartphone Addiction Scale (SAS) to enable a more nuanced understanding of problematic use.

A further limitation of this study is the exclusive reliance on self-reported data. Both smartphone usage and mental health indicators were assessed via participant self-report, which may introduce measurement bias. Participants might underreport or overreport their behaviors due to recall inaccuracies or social desirability concerns. While the mental health scales used have strong psychometric properties, future studies should consider combining self-reports with objective measures, such as digital usage tracking or clinician-based assessments, to improve data accuracy.

Another potential limitation stems from the online survey methodology. Although the sampling strategy ensured representativeness across major sociodemographic dimensions, individuals with limited internet access or digital literacy—particularly older adults or residents of rural areas—may have been systematically underrepresented. This may introduce sampling bias and could influence observed associations between smartphone use and sociodemographic factors. Future studies should consider mixed-mode survey approaches or targeted oversampling to better reach digitally less-connected populations.

Furthermore, our analytical approach was limited to assessing direct associations. More advanced statistical modeling, such as mediation or moderation analyses or structural equation modeling (SEM), was beyond the scope of this study but could provide valuable insights into causal mechanisms in future research.

A further limitation is the unequal sample size between the two survey waves (*n* = 1,031 in 2022 vs. *n* = 2,021 in 2024), which may affect the comparability of results across time points.

## 5 Conclusion

The study highlights a significant increase in smartphone usage between 2022 and 2024 in Austria, particularly among adults aged 25 and older, with problematic use (at least 3 h daily) strongly associated with mental health issues such as depression, anxiety, insomnia, alcohol abuse, and high stress. Women, younger individuals, and urban residents, especially in Vienna, were identified as high-risk groups for excessive use. These findings emphasize the need for interventions promoting digital well-being through targeted strategies such as digital literacy programs, mental health screenings, and approaches to curb excessive smartphone dependency. Given the cross-sectional nature of the study, future research should adopt longitudinal designs to establish causal pathways and explore the impact of qualitative aspects of smartphone use.

## Data Availability

The raw data supporting the conclusions of this article will be made available by the authors, without undue reservation.
